# Making sense of the evidence: designing randomised controlled trials for preterm infants with high-shunt volume patent ductus arteriosus

**DOI:** 10.1136/archdischild-2025-330266

**Published:** 2026-04-27

**Authors:** Cheryl Battersby, Philip T Levy, Samir Gupta, Willem P de Boode, Souvik Mitra, Patrick J McNamara

**Affiliations:** 1 Neonatal Medicine, School of Public Health, Faculty of Medicine, Imperial College London, London, UK; 2 Centre for Paediatrics and Child Health, Imperial College London, London, UK; 3 Boston Children's Hospital, Harvard Medical School, Harvard University, Boston, Massachusetts, USA; 4 Department of Engineering, Durham University, Durham, UK; 5 Division of Neonatology, Department of Paediatrics, Sidra Medicine, Doha, Qatar; 6 Department of Neonatology, Amalia Children's Hospital, Raboud University Medical Center, Nijmegen, Netherlands; 7 Division of Neonatology, University of British Columbia, Vancouver, British Columbia, Canada; 8 Division of Neonatology, Department of Paediatrics, University of Iowa, Iowa City, Iowa, USA

**Keywords:** Neonatology, Child Health, Cardiology, Intensive Care Units, Neonatal, Physiology

## Abstract

Management of the patent ductus arteriosus (PDA) in preterm infants remains controversial. Randomised controlled trials (RCTs) have shown that administering pharmacotherapy, predominantly ibuprofen, a cyclooxygenase (COX) inhibitor, to infants selected based on the standard echocardiography approach to diagnosis (eg, duct diameter and shunt direction), does not improve outcomes and may lead to harm. It remains uncertain, however, whether eliminating or reducing PDA shunt volume using an intervention with higher efficacy and fewer adverse effects, or in a more selective population, would show different results. It is possible that an imprecise approach to patient selection exposes low-risk infants to the adverse effects of pharmacotherapy without benefit and high-risk infants to the synergistic adverse effects of pharmacotherapy and persistent high volume pathological shunt when treatment fails. Whether targeted management of moderate-high volume PDA shunts, informed by comprehensive echocardiography adjudication, in the highest risk infants is beneficial remains untested in an RCT setting. Furthermore, both pharmacological and non-pharmacological interventions warrant further investigation. High quality practice changing research requires a collaborative approach between haemodynamic specialists, epidemiologists and trial methodologists to (1) define the study population based on phenotypic profiles of high-risk infants; (2) enhance the choice and timing of intervention; and (3) identify outcome measures that are relevant and clinically meaningful to families. In this review, we summarise evidence from RCTs and observational studies by discerning discrepancies and exploring potential explanations. Such an approach is essential to establish whether active PDA treatment confers any measurable benefit for high-risk preterm infants.

## Introduction

 Management of patent ductus arteriosus (PDA) in preterm infants is controversial, with uncertainty regarding whether, when, how and in whom to intervene. Physiological and epidemiological studies support the association between PDA and increased mortality and morbidity.^1^ Recent meta-analyses of randomised controlled trials (RCTs) of ibuprofen <72 hours have not demonstrated benefit and concerningly an increased risk of mortality.^2 3^ On the contrary, results from a single centre observational study suggest that early echocardiography screening (<24 hours) where PDA treatment is targeted to moderate-high volume shunts, and phenotypes where the PDA is beneficial (eg, heart dysfunction, pulmonary hypertension) are excluded, may confer benefit.^4^ The high rates of spontaneous closure, unpredictable efficacy and potential adverse effects of PDA pharmacotherapy further complicate decision-making.^1 2^

Accordingly, opinions remain divided on whether active PDA treatment alters outcomes in preterm infants^2 3^ and whether PDA presence is merely a biomarker for worse outcomes in the most immature patients.^1^ Meanwhile, a growing number of studies have shown similar or increasing rates of bronchopulmonary dysplasia (BPD).^5^ It is uncertain whether these changes relate to increased survival at lower gestational ages (GAs), a decline in active PDA management, or non-judicious use of cyclooxygenase (COX) and peroxidase (POX) inhibitors. In this review, we provide an interpretation of the current evidence from RCTs and observational studies, outline ongoing areas of uncertainties and propose priorities for future investigation.

### RCT evidence

#### ‘Early’ active pharmacotherapy treatment of PDA within the first week

**Figure 1:**
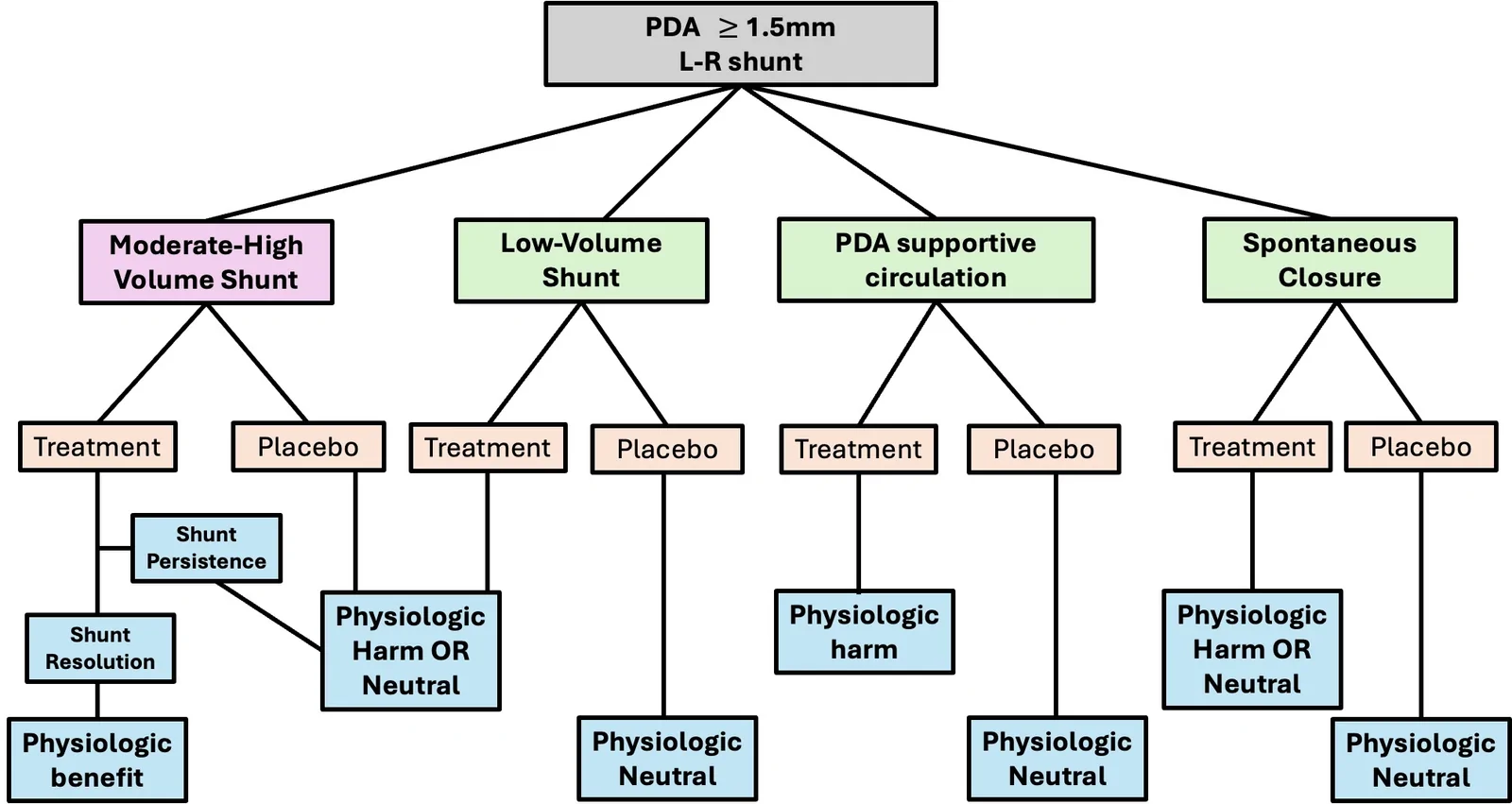
PDA phenotypes for a PDA >1.5 mm (reproduced with permission from Bischoff *et al*^8^). PDA, patent ductus arteriosus.

The recent 2025 Cochrane review (19 RCTs, 2323 participants) reported that early (within the first 7 days) active pharmacotherapeutic treatment of a PDA compared with an expectant approach results in no difference in mortality in preterm infants (moderate-certainty evidence) and no difference in BPD, severe intraventricular haemorrhage (IVH) or necrotising enterocolitis (NEC) (low-certainty to moderate-certainty evidence).^2^ Of note, the inclusion criteria were broad (<37 weeks or <2500 g) and may not be reflective of findings in the most extremely preterm infants. Of the most recent and largest published trials of very early (<72 hours) management, Baby-OSCAR (Outcome after Selective early treatment for Closure of patent ductus ARteriosus)^6^ showed no difference between very early ibuprofen therapy versus placebo in the primary composite outcome of death or moderate/severe BPD, while Early Treatment Versus Expectative Management of PDA in Preterm Infants (BeNeDuctus) trial^7^ demonstrated non-inferiority of an expectant management approach versus very early ibuprofen therapy with a primary composite outcome of NEC, moderate/severe BPD or death. Overall, infants in the early treatment group of the BeNeDuctus trial fared worse, with a higher rate of the composite primary outcome compared with expectant management, mainly driven by increased incidence of moderate-severe BPD. Of concern, there was higher mortality among extremely preterm infants exposed to very early ibuprofen (48–72 hours). A further meta-analysis included trials of PDA treatment up to 14 days of age, also showed no benefit (and possible harm) between active as compared with expectant management.^3^ The authors of the Cochrane review acknowledged the need for caution given the limited echocardiography eligibility criteria, predominantly based on PDA diameter or shunt direction, which are not direct quantifiers of shunt volume.^2^ In addition there is increasing evidence that the biological role of the PDA in the early transitional period is complex with many distinct phenotypes that may not have been delineated (figure 1).^8^ The review also acknowledged that the impact of selective intervention in the first 24 hours on IVH and pulmonary haemorrhage (morbidities which usually occur in the first 72 hours) has not been tested in a large RCT. While the DETECT (Ductal Echocardiographic Targeting and Early Closure) trial^9^ demonstrated a lower rate in pulmonary haemorrhage and a trend towards a lower IVH rate after very early (<12 hours) indomethacin; of note, these differences were not observed in the BeNeDuctus and BabyOSCAR randomised trials which may relate to the timing of intervention. In addition, the DETECT trial was underpowered.

#### ‘Later’ active pharmacotherapy treatment of PDA beyond the first week

Administering ‘later’ active pharmacotherapy (days 7–14) avoids exposing infants who might experience early spontaneous PDA constriction to unnecessary intervention. This approach is not without risk, as the potential adverse effects of pharmacotherapy on the immature lung may be exaggerated in patients with prolonged PDA exposure.^5^ The PDA-TOLERATE (TO LEave it alone or Respond And Treat Early) trial randomised infants born 23–27^+6^ weeks gestation at age 6–14 days. It showed no differences in either the primary outcome of PDA ligation or presence of PDA at discharge between the intervention (indomethacin/ibuprofen/acetaminophen at clinician’s discretion) or control groups (conservative management).^10^ Infants in the treatment group compared with controls were exposed to a moderate-to-large PDA for a shorter duration after randomisation (median, 7.5 days vs 22 days). Caution in interpretation is warranted, as patient enrolment was based on a limited definition of haemodynamic significance and the rate of open-label treatment was high in the control group (48%). Similarly, the Neonatal Research Network (NRN) PDA RCT, where infants born 22–28 weeks were randomised between 48 hours and 21 days (median postnatal age 10 days), also demonstrated no difference in the primary outcome of death or BPD at 36 weeks between active (acetaminophen, ibuprofen or indomethacin) and expectant treatment groups.^11^ The finding of an increase in mortality in the ‘active treatment’ group is a concern. Importantly, both trials raise questions regarding generalisability. In PDA-TOLERATE, the non-enrolled population were younger, sicker and more likely to receive earlier pharmacotherapy; interestingly, the rate of death and BPD were lower in these patients than those enrolled in the trial. In the NRN PDA trial, the sickest infants with cardiopulmonary compromise were excluded due to lack of clinician equipoise; specifically, two-thirds of eligible but non-enrolled infants received pharmacological treatment. Collectively, these findings underscore a critical knowledge gap: whether these results are applicable to infants with high volume, haemodynamically significant PDA shunts remains uncertain. This population, arguably at highest risk of PDA-related morbidity requires rigorous prospective evaluation.

### Transcatheter PDA closure

Advances in transcatheter device closure of the PDA have led to increasing adoption among centres with access to interventional cardiology expertise.^12^ While promising results, there remains a need to evaluate whether transcatheter PDA closure improves short- and longer-term outcomes and the optimum timing.^1^ The ongoing Percutaneous Intervention Versus Observational Trial of Arterial Ductus in Low weight Infants (PIVOTAL, NCT05547165) will help contribute to the evidence base; however, some enrolled infants may have received pharmacotherapy prior to randomisation, which is a potential confounder.

### Interpretations of RCT evidence

- We may infer from early intervention trials that ibuprofen use for infants enrolled based on PDA diameter >1.5 mm and shunt direction as the primary criterion is not beneficial and potentially harmful. Whether these findings are applicable to infants with moderate-high volume shunts, selected based on multiparametric echocardiography and randomised to a treatment pathway that guaranteed shunt resolution and persistence in the expectant arm remains to be tested.
- Published literature demonstrates that PDA diameter has poor predictability of spontaneous closure^8^ and interobserver reproducibility.^13^ Active treatment solely based on PDA diameter and shunt direction can result in up to one-third of low-risk infants being exposed to the adverse effects of pharmacotherapy.^13^ Data from a natural history dataset of 357 extremely preterm infants demonstrated that 50% of infants between 21–27 weeks gestation have a PDA diameter >1.5 mm, of whom only 40% had a moderate-high volume shunt on multiparametric echocardiography evaluation.^8^ Of concern, 30% patients had PDA phenotypes (eg, pulmonary hypertension, heart dysfunction) where treatment may be harmful.
- The signal of harm seen with early ibuprofen use in RCTs^2^ is an important consideration. There is animal experimental evidence that ibuprofen could have harmful effects on lung angiogenesis.^14^ On the contrary, data from the preterm baboon model demonstrated improved lung compliance, reduced lung injury and enhanced alveologenesis.^15^ No data on pulmonary vasculogenesis were presented in this study. Data from the Trial of Indomethacin Prophylaxis in Preterms (TIPP) trial demonstrated a higher rate of BPD (43% vs 30%) in patients with no PDA who received indomethacin treatment lends credence to the concern about non-judicious use of pharmacotherapy.^16^
- The efficacy of pharmacotherapy is variable with declining effectiveness of each subsequent course. Treatment success could be affected by lower GA, higher PDA severity scores, diastolic dysfunction, systemic hypoperfusion and altered myocardial strain patterns.^1^ We may therefore risk exposing a subset of infants to drug toxicity without achieving the therapeutic goal. Further research is needed to explore safer dosing strategies, alternative pharmacotherapy or non-pharmacotherapy options with favourable risk-benefit profiles across different gestational ages.^1^
- Transcatheter device closure offers benefits over surgical ligation, but the selection criteria and optimal timing remain to be determined.^12^
- Although acetaminophen use is rising,^1^ conflicting findings from human versus animal experimental studies highlight the importance of prospective evaluation in an RCT setting. Preclinical and human data suggest that acetaminophen may be harmful to the developing lung,^1^ described as induced cell damage from the conversion of acetaminophen to the toxic metabolite N-acetyl-p-benzoquinone imine. While these concerns are legitimate, evidence from the TREOCAPA trial (TReatment with Early Oral CAcetaminoPA, acetaminophen), conducted in 43 neonatal intensive care units (NICUs) across Europe, showed no increased BPD in the prophylactic acetaminophen group.^17^ Of note, while selective acetaminophen use in the first 24 hours for moderate-high volume shunts showed lower rate of severe BPD in infants <24 weeks,these findings were not replicated in more mature infants.^4^

### Interpretations of evidence from epidemiological and observational studies

Observational studies evaluating the impact of early targeted PDA management report inconsistent findings. A selection of observational studies published in recent years are presented with an assessment of the risk of bias using the ROBINS-I (Risk Of Bias In Non-Randomized Studies–of Interventions) tool^18^ in table 1 and online supplemental file 1.^4 19–25^ It is noteworthy that in studies showing no benefit or even harm from pharmacotherapy, the diagnosis of PDA was made on the basis of diameter and shunt direction alone. On the contrary, data from one centre, where very early acetaminophen (<24 hours) was targeted to a highly select population of moderate-high volume shunts, identified using multiparametric echocardiographic assessment (indices of pulmonary overcirculation, left ventricular volume overload, diastolic function and systemic hypoperfusion), reported improved post-interventional outcomes despite the bias of a higher proportion (44%) of infants <24 weeks in the haemodynamic epoch.^4^ It is plausible that these outcomes relate to enhanced characterisation of PDA phenotypes and distinguishing PDA supportive circulations (treatment was avoided) from pathological shunts (treatment was considered). Although these findings are hypothesis generating, they are meaningful to clinicians, who strive to enhance outcomes of the most vulnerable preterm infants, and warrant testing in an RCT setting. There are, however, inherent limitations to observational studies:

- Risk of bias inherent to observational studies (online supplemental file 1). Although appropriate statistical methodology has been used to account for known confounders (including logistic regression, propensity matching and instrumental variables), causality cannot be attributed to the exposure.
- Before/after study designs are subjected to confounding due to secular changes in clinical practices over time and baseline population differences. Statistical adjustments cannot consider unmeasured or unknown confounders.
- Many of the observational studies are single or smaller multicentre studies, which limits generalisability.^4 19 25^ For example, if the treatment-failure cohort group in one of the studies is small, statistical power and generalisability will be further reduced.
- The findings and applicability may not be replicable in centres without neonatal haemodynamic expertise.

**Table 1:** Selected observational studies evaluating the impact of early screening echocardiography and targeted patent ductus arteriosus treatment in preterm infants

Author, Country Aim	Method Study design, inclusion population	Criteria for hsPDA Management strategy	Key findings
Rozé *et al*,^19^ France To test the hypothesis that early screening echocardiography for PDA would be associated with more frequent PDA treatment and better outcome	National, prospective, population-based cohort study in France, conducted year 2011. Infants 24–28 weeks. Comparison groups Exposed to early echocardiogram <3 days (n=605). Not exposed to early screening echocardiography (n=605). Primary outcome: Observational on PDA diagnosis and characteristics, common neonatal morbidities (NEC, BPD, pulmonary haemorrhage, mortality).	**Threshold for treatment:** Ductus arteriosus diameter >1.5 mm/kg. Pulsatile ductus shunt flow pattern. High-velocity flow in left pulmonary artery. Diastolic aortic flow reversal or low superior vena cava flow. Information was collected about PDA diagnosis, including markers from the echocardiogram (ductus arteriosus diameter >1.5 mm), high-velocity flow in the left pulmonary artery (mean velocity >0.4 m/s or end-diastolic velocity >0.2 m/s), diastolic aortic flow reversal or low superior vena cava flow (<40 mL/kg/min), pulsatile ductus shunt flow pattern, date of treatment with ibuprofen or surgical ligation and indication for treatment.	**Exposed infants** Treated for PDA more frequently during their hospitalisation. Lower hospital death rate. Lower rate of pulmonary haemorrhage. No differences in rates of NEC, BPD, severe bronchopulmonary dysplasia or severe cerebral lesions were observed.
Okulu *et al*,^20^ Turkey Prospective online registry database to examine the variations in PDA management for a nationally-based cohort, and evaluate the effects of PDA management strategies on the rates of PDA closure, PDA ligation, associated morbidities and survival in preterm infants. Compare conservative vs medical treatment in preterm infants with moderate-to-large PDA	Prospective, multicentre, observational cohort study of infants between 24 0/7 and 28 6/7 weeks Echocardiograms were obtained in the first 3 weeks of age (median of 3 days with IQR of 2 days) and the decision for management was left up to each centre according to their standard of practice. Two groups: Infants with moderate-to-large PDA were grouped according to their PDA management as: Conservative approach. Medical treatment. Primary outcome: Associated morbidities, mortality rate, and BPD or death (BPD/death) were compared between the two groups.	Diagnosis of PDA was based on clinical and/or echocardiographic studies, which included two-dimensional imaging, M-mode, colour flow mapping and Doppler interrogation. A moderate-to-large PDA was defined as an internal ductus diameter >1.5 mm, left atrium-to-aortic root ratio >1.5 and reverse diastolic flow in the descending aorta. A ductus that failed to meet these criteria was defined as small PDA.	Among the 1193 enrolled infants (26.7±1.4 weeks and 926±243 g), 544 (46%) had moderate-to-large PDA. 130 (24%) infants with moderate-to-large PDA were managed conservatively, in contrast to 414 (76%) who received medical treatment. Medical treatment had no effect on the incidence of surgical ligation or prematurity-related morbidities in the overall study group. Infants treated before postnatal day 7 had higher rates of mortality and BPD/death than infants who were conservatively managed or treated beyond day 7 (p=0.009 and 0.007, respectively). The preferred treatment options were ibuprofen and paracetamol as the initial course; indomethacin was not used in any patient. Medical treatment had no effect on the incidence of surgical ligation or prematurity-related morbidities in the overall study group.
Chen *et al*,^21^ China Assess risk of BPD with ibuprofen-treated PDA closure	Single centre, retrospective cohort; all neonates born less than 28 weeks’ gestation. An echocardiogram was routinely performed within 72 hours after birth. Two groups: Infants received ibuprofen if they had an hsPDA. Infants did not receive pharmacotherapy if they did not have an hsPDA. Primary outcome: BPD was diagnosed when supplemental oxygen was required at 36 weeks PMA or discharge.	Presence of a ductus with a diameter >1.5 mm, left atrial inner diameter/aortic root (LA/AO) ≥1.4, combined left to right shunt. Preterm infants with PDA—treated with ibuprofen for closure (diagnosis presumably by echo+clinical criteria). Medical closure using ibuprofen.	BPD is approximately twofold more likely to occur in infants exposed to ibuprofen during the first days of life.
Termerova *et al*^22^ Czech Republic Compare early targeted ibuprofen vs expectant management in extremely preterm infants	Single centre, retrospective cohort; all viable infants born between 23 0/7 and 27 6/7 weeks. All infants screened by echocardiography in the first 23 days of age. Two groups of infants. Early ibuprofen (first 3 days). Expectant management (treatment only if symptomatic). The primary outcome of this study was the composite outcome of BPD and/or death before 36 week PMA.	Definition of hsPDA was based on the diameter of PDA of at least 2 mm and pulsatile flow pattern measured in the high left parasternal view, the site of maximal constriction with colour images, left-to-right flow from hsPDA and high-risk clinical factors (no antenatal steroids, difficult adaptation after delivery, or high ventilation support in the first days of life). Early targeted epoch from 2011 to 2013 with hsPDA based on echocardiographic and clinical features received Ibuprofen <3 days of age. Expectant treatment period (2013–2015) were observed and not treated in first 3 days of age.	Early targeted ibuprofen associated with higher incidence of death or severe BPD, no reduction in NEC, IVH, or ROP; expectant management had fewer adverse outcomes; suggests potential harm of early routine treatment.
Terrin *et al*,^23^ Italy Evaluate echocardiography-guided management of PDA regardless of symptoms in VLBW infants^23^	Single centre, prospective cohort; preterm infants <32 weeks gestation or birth weight <1500 g with echocardiographically diagnosed PDA. Two arms: Immediate pharmacological closure of hsPDA (treat all). Pharmacological therapy only if clinical+echocardiographic signs of instability—defined as clinical signs of haemodynamic instability including acidosis, increased refill capillary time (evaluated three times consecutively), increased oxygen requirement, oliguria and persistent hypotension (evaluated within 12 hours). (physiology guided) Primary outcome: Rate of newborns who survived without any morbidities on discharge from NICU. Morbidity was defined as the presence of at least one of the following conditions occurring after enrolment: NEC Bell stage ≥2, IVH stage ≥2 (observed after 72 hours of life), retinopathy of prematurity stage ≥2, BPD, periventricular leukomalacia, prolonged mechanical ventilation (more than 7 days consecutively) and acute renal failure.	Haemodynamically significant PDA defined by echocardiography parameters (ductal diameter, LA/Ao ratio, flow pattern). Pharmacological treatment with ibuprofen was the first therapeutic choice for PDA treatment.	Cohort A (treat all) had significantly higher survival without morbidity (48.1% vs 22.2%); lower rate of IVH ≥stage 2 in treat all vs guided cohort (3.7% vs 24.4%).
Isayama *et al*^24^ iNeo network Evaluate presymptomatic PDA treatment based on early routine echocardiography, performed regardless of clinical signs, improved outcomes	Retrospective, multicentre, survey-linked cohort study used an institutional-level questionnaire and individual patient-level data and included infants of <29 weeks gestation of 9 population-based national or regional neonatal networks; 17 936 infants <29 weeks GA across 246 NICUs. Two groups: Infants in NICUs receiving early screening by routine echocardiography+presymptomatic treatment echocardiography. Infants who did not receive presymptomatic PDA treatment. Primary composite outcome (early death or severe intraventricular haemorrhage) and secondary outcomes (any in-hospital mortality and major morbidities.	NICU-level early screening+presymptomatic treatment vs centres not treating presymptomatic PDA.	The primary outcome of early death or severe intraventricular haemorrhage was not significantly different between the NICUs treating presymptomatic PDA and those who did not (17% vs 21%; aOR 1.00, 95% CI 0.85 to 1.18) higher odds of ROP treatment in treated centres; routine presymptomatic treatment not associated with clear benefit no uniform hsPDA definition across units.
Giesinger *et al*^4^ Iowa, USA Compare impact of early hsPDA management based on TnECHO evaluation in extremely preterm infants born at 22^+0^ to 23^+6^ weeks GA and 24–26 week infants, as compared with a historical epoch during which exclusively clinically ‘symptomatic’ treatment after postnatal day 7^4^	Single centre, retrospective. Infants <27 weeks, diagnosed with hsPDA. **Two epochs:** Haemodynamic screening epoch (2018–2022, n=181). Historical controls epoch (2010–2017, n=320). **Primary outcome**: Composite of survival-free from grade 3 BPD for birth year.	**Haemodynamic screening epoch:** TnECHO performed by neonatologist with specialised training in echocardiography at 12–18 hour postnatal age. **Threshold for treatment: Iowa PDA score ≥6** First-line acetaminophen 15 mg/kg IV 6 hourly for 12–16 doses. Repeat TnECHO after 12th dose, if PDA score ≥6 complete 28 total doses. Indomethacin was used as second line ×3 doses, 1–2 trials. Shunt modulation strategies: avoidance of anaemia and hypocapnaemia, tolerance of low target sats 85–90%. If remains significant both clinically and haemodynamically, for definitive closure by percutaneous device. **Historical cohort epoch**: Echocardiograms were performed by paediatric sonographers and interpreted by cardiologists. **Threshold for treatment:** PDA diameter >1.5 mm with left to right shunt Standard of care to conduct echocardiogram on day 6 or 7 to screen for PDA. Follow-up scans only when signs of hsPDA judged to be present. Management was using three courses of indomethacin followed by surgical ligation if remained significant.	**Haemodynamic screening epoch:** Found a 50% reduction (p<0.002) in the primary composite outcome of death before 36 weeks or grade 3 BPD in the 22^+0^ to 23^+6^-week patients. Increase (p=0.002) in survival free of severe morbidity to 73%. Reductions in the rate of severe IVH (p=0.001). No cases of pulmonary haemorrhage, a non-significant but relevant trend of an already very low rate. In the older cohort 24–26 weeks, there was no difference in the primary outcome or rates of BPD; there was an increase in survival-free of severe morbidity (p<0.001) and major reductions in the rate of NEC (p=0.01) and severe IVH (p<0.001). No long-term follow period beyond the neonatal period.
Mullaly *et al*^25^ Dublin, Ireland Evaluate whether early targeted risk-based management of a PDA improves outcomes in preterm infants?	Single centre, observational cohort study. Infants born <29 weeks’ gestation. **Comparison groups (2020–2024**) High-risk based on El-Khuffash PDAsc, (n=74). Low-risk PDAsc (n=110). Note: High-risk infants in the early-targeted treatment epoch received medical intervention, while low-risk infants did not. **Additional comparison groups (<2015**) Primary outcomes from the early risk-based screening epoch were compared with a historical reference epoch in which risk was assigned but treatment was not implemented. Low risk n=51. High risk n=84.	**Threshold for treatment:** Deemed high risk if PDAsc<5. Received ibuprofen course. If remained open, received a second ibuprofen course with the same dosing schedule. If ibuprofen contraindicated, then paracetamol was used. If PDA persisted, a second paracetamol course was given If PDA persists after two courses of medical treatment and continues to require intubation and ventilation, referral for PDA device closure. Infants with a score of <5 were categorised as low risk and were not treated. For this low-risk cohort, an echo was repeated at day 14.	**High-risk infants in the early-targeted treatment epoch** Successful PDA closure following treatment: Improvements in respiratory outcomes, including fewer ventilation and oxygen days, as well as a lower incidence of CLD. Treatment-failure group Outcomes comparable to those of the reference epoch, highlighting the fact that PDA management is perhaps more complex in this subgroup. **Low-risk infants** Decision to not treat their PDA was effective, with consistently favourable outcomes and minimal interventions implemented across both epochs.

aOR, adjusted OR; BPD, bronchopulmonary dysplasia; CLD, chronic lung disease; GA, gestation age; hsPDA, haemodynamically significant PDA; IV, intravenous; IVH, intraventricular haemorrhage; LA/Ao, left atrium-to-aortic root ratio; NEC, necrotising enterocolitis; NICU, neonatal intensive care unit; PDA, patent ductus arteriosus; PDAsc, PDA Severity Score; PMA, postmenstrual age; ROP, retinopathy of prematurity; VLBW, very low birth weight.

On the contrary, unlike RCTs which may only recruit a proportion of eligible infants and hence generalisability is limited, observational studies represent a ‘real-world’ experience which is a strength.

### Considerations for future steps

Key research questions:

- In infants born less than 28 weeks GA with a moderate-to-high volume PDA shunt (POPULATION), does an active PDA management pathway commencing *less than 24 hours* (VERY EARLY INTERVENTION) compared with truly expectant pathway (CONTROL) reduce the risk of IVH, pulmonary haemorrhage, moderate-severe BPD and mortality?
- In infants born less than 28 weeks GA with a moderate-to-high volume PDA shunt (POPULATION), does an active PDA management pathway commencing between 7–14 postnatal days (LATER INTERVENTION) compared with truly expectant pathway (CONTROL) reduce moderate-severe BPD, duration of mechanical ventilation and non-invasive ventilation, and mortality?

### Population selection

- Infants less than 28 weeks GA have the highest risk of PDA attributable morbidity.
- Multiparametric echocardiography adjudication to facilitate enrolment of infants with a ‘moderate-high volume’ shunt.
- Expert panel to appraise evidence and develop recommendations for incorporation of multiparametric echocardiography scores into RCTs. Numerous PDA scores have been developed (table 2),^26–30^ but their concordance has not been studied. While some have been validated against BPD,^26 28^ the suitability of this endpoint—given the diversity of the underlying phenotypes in BPD—is questionable. In addition, the relationship of PDA scores to other relevant morbidities (IVH, pulmonary haemorrhage) has been poorly studied.^29^

**Table 2:** Patent ductus arteriosus scoring systems used across programmes worldwide

First author, PDA score	Study methodology (design, population, statistical approach, validation comparator)	Key findings/conclusions	PDA score parameters and scoring approach
McNamara and Sehgal^36^	**Study design and population** Consensus/expert-derived staging system based on prospective clinical/echo observations in preterm infants. <32 weeks gestational age. **Score validated against:** Higher composite scores associated with increased risk of developing of CLD. Validated against clinical diagnosis of haemodynamically significant PDA and used widely in observational and interventional studies. **Statistical analyses**: No regression models.	**Key findings** Proposed staging system for determining the magnitude of the haemodynamically significant ductus arteriosus which is based on clinical and echocardiographic criteria. **Conclusion** Future clinical trials of treatment should be less pragmatic but more focused with strict inclusion criteria. First study to introduce the concept of staging PDA severity using integrated echocardiographic and physiological findings, rather than ductal size alone.	**Clinical** (mild, moderate or severe) Pulmonary overdilation Oxygenation index. Ventilatory requirements. Systemic hypotension Oliguria/renal function. Metabolic acidosis. NEC-like abdominal distension. **Echocardiography** Transductal diameter. Transductal flow. LA/Ao ratio. E/A ratio. IVRT. Flow in the superior mesenteric artery, middle cerebral artery or renal artery. **Scoring:** 0–3 points in each domain; higher scores indicate greater haemodynamic significance. Categorical staging (mild–moderate–severe); higher stage=more significant PDA.
Elsayed and Fraser *et al*, Manitoba PDA score 2012^37^	**Study design and population** Prospective single centre study presented as an abstract in Winnipeg, Canada. Infants <31 weeks (2010–2011). Echocardiogram performed between 2–3 days of age. Utilisation of PDA score. Published in abstract form. **Score validated against:** compared with a PDA diameter >1.5.	**Key findings** Both the clinical and echo component correlated strongly with each other. The PDA score and components significantly predicted hsPDA. PDAsc at day 2 strongly predicted CLD/death, outperforming single echo indices. **Conclusions** The Manitoba PDA score performed at 48–72 hours of age predicts hsPDA who eventually received treatment. Use of PDA score may reduce the number of infants who are treated with non-significant PDA.	**PDA scoring system** Clinical and echocardiographic measures of volume and pressure overload. PDA diameter indexed to weight. LA:Ao ratio. Ductal flow pattern. Systemic diastolic flow changes. **Scoring:** The PDA score is a numerical score (maximum 28) incorporating echocardiographic parameters reflective of both volume and pressure overload (max score 15), and clinical, radiological and laboratory features of both pulmonary over-circulation and systemic hypoperfusion (max score 13).
El-Khuffash *et al*, El-Khuffash PDA Severity Score^13 25 26 38^	**Original study design and population** Ireland, Canada and Australia.^25 26 38^ Prospective observational study. 141 infants born <29 weeks’ gestation. Echocardiogram done three time points: median 10 hours (day 1), 43 hours (day 2), 144 hours (5–7 days). Echocardiogram performed on postnatal age day 2 to measure PDA diameter and maximum flow velocity, LV output, diastolic flow in the descending aorta and coeliac trunk and variables of LV function using tissue Doppler imaging. Predictors of CLD/death were identified using logistic regression methods. **Score validated against**: Receiver operating characteristic curve was constructed to assess its ability to predict CLD/death. Validated internally. **Statistical approach:** Predictors of CLD/death were identified using logistic regression method. **Follow-up observational study design and population** Single centre retrospective observation study of prospectively acquired data. Compare outcomes between high-risk and low-risk infants from an early-targeted treatment epoch (n=184) and a historical reference epoch (n=135) who were risk stratified but did not undergo treatment. Used El-Khuffash PDA Severity Score to classify risk.	**Key findings** Original study and RCT Five variables independently associated with CLD/death (gestation at birth, PDA diameter, maximum flow velocity, LV output, LV a’ wave. PDAsc range 0 (low risk) to 13 (high risk). PDAsc cut-off of 5 had sensitivity and specificity of 92% and 87%, and positive and negative predictive values of 92% and 82%, respectively. Follow-up observational study High-risk infants in the early-targeted treatment epoch who achieved successful PDA closure demonstrated fewer ventilation days, fewer oxygen days and a lower incidence of CLD compared with high-risk infants in the reference epoch. **Conclusions** Using a PDAsc for infant recruitment to a PDA treatment randomised controlled trial is feasible. There is a high rate of consent and relatively low rate of open-label PDA treatment. The implementation of early-targeted risk-based PDA management may be associated with improved respiratory outcomes in high-risk infants who successfully responded to treatment.	**PDA scoring system** The following equation was used to derive the risk score for each infant: (gestation in weeks × −1.304) + (PDA diameter in mm × 0.781) + (LVO in mL/kg/min × 0.008) + (maximum PDA velocity in m/s × −1.065) + (LV a’ wave in cm/s × 0.470) + 41, where 41 is the constant of the formula. **Scoring:** This score ranges between 0 (low risk) and 13 (high risk). The predicted probability for each infant to develop CLD/death was also derived from the model.
Fink *et al* The SZMC PDA Severity Score (2017)^28^	**Study design and population** Institutional score applied in clinical cohort studies at SZMC. Aim was to assess the ability of the SZMC to evaluate haemodynamic significance of the PDA and similarly predict outcome of CLD as El-Khuffash score. **Score validated against:** Composite outcome of chronic lung disease/death and PVL, correlation with El-Khuffash PDAsc.	**Key findings** Comparison showed two scores were well correlated with each other and both scores positively predicted chronic lung disease/death in this population. El-Khuffash score correlated. Significantly with NEC, the SZMC score did not. **Conclusions** Closely correlated with PDAsc; higher score associated with increased risk of adverse outcomes, including white matter injury. Stepwise progression in the incidence of poor outcome parameters based on PDA score. Both the El-Khuffash and SZMC scores are predictive of PDA-associated morbidities.	**PDA scoring system** Ductus arteriosus diameter. LA/Ao ratio. Retrograde diastolic flow in the abdominal aorta diastolic flow. Ductus arteriosus shunt flow pattern from the high left parasternal short-axis view. **Scoring**: Scores for each between 0–2. A composite score of 6 is considered haemodynamically significant; a score of 3–5 is considered borderline significance; and a PDA with a score of 0–2 is considered haemodynamically insignificant.
Rios *et al*. Iowa Score^4 27^	**Study design and population** Toronto single-centre 2011–2014. Single centre (Toronto) retrospective study. 137 neonates born at <32 weeks’ gestation. TnECHO evaluation of PDA performed between 7 and 30 days (median of 18 days, IQR, 16–19 days). Validated within the University of Iowa TnECHO programme. **Score validated against**: Composite of individually validated markers (increased risk of ventilator requirement and composite outcomes of death and CLD). **Statistical approach:** A univariate Poisson regression analysis was conducted to identify the correlation of each individual echocardiographic parameter with descending aorta holodiastolic flow reversal.	**Key findings** The best correlation was observed with descending aorta and coeliac artery diastolic flow reversal. Values for the pulmonary vein D-wave peak velocity, mitral E-wave peak velocity, LA: Ao ratio, LVEDD and LVO were highest and IVRT shortest in the group of PDA with holodiastolic flow reversal. **Conclusion**: A more comprehensive assessment using multiple parameters may provide a more holistic appraisal of the shunt volume and haemodynamic significance. The accuracy of the assessment of the shunt volume is likely to be increased when multiple measurements reach target threshold values.	**PDA scoring system** Pulmonary vein D wave. Peak mitral valve E wave velocity. Isovolumetric relaxation time. LA/Ao. Left to right ventricular output ratio. Aortic/peripheral doppler flow reversal. Ductus diameter indexed to weight (mm/kg). **Scoring:** Scores for each between 0–2, A score of 6 or above is considered haemodynamically significant (as determined by expert consensus).
Umapathi *et al* PDA Severity Score^39^	**Study design and population** Chicago (Rush Medical Center). Prospective single centre observational study. Develop a PDA score with direct measures pulmonary blood flow. 124 babies >31 weeks GA at delivery. The median (IQR) time of echocardiography was 54 hours (39–70). **Score validated against:** CLD/death. **Statistical approach:** Multivariate logistic regression analysis to derive the PDA score. Compared the predictive accuracy of PDA Score against PDA diameter, LA:Ao ratio and DFR independently.	**Key findings** Echocardiography variables suggestive of either pulmonary over perfusion or systemic hypoperfusion along with measurements of LV diastolic function obtained within 1 week of birth. A weighted scoring system based on the beta coefficients of the significant predictors was used to derive the PDA score. The following equation was used to calculate the risk score for each infant. **Conclusions:** PDA severity score of 5.5 has a sensitivity and specificity of 94% and 93%, and positive and negative predictive values of 94% and 93%, respectively.	**PDA scoring system** LPA diameter (mm). LPA systolic and diastolic VTI in cm. LPA flow (L/min/m^2^). RPA diameter (mm). RPA systolic and diastolic VTI (cm). RPA flow (L/min/m^2^). PDA diameter (mm), PDA velocity (m/s), mitral inflow velocities E wave (m/s). A wave (m/s) and E/A ratio. PV V_d_ in pulmonary vein (m/s). DFR (%), systolic and diastolic flow VTI in SMA and coeliac artery (cm). LVO (mL/kg/min), ratio of left atrial size to aortic root diameter (LA to AO ratio). **Scoring:** The score ranges from 0 (low risk) to 12 (high risk).
Masutani *et al*., PLASE score (2024)^40^	**Study design and population** Post-hoc analysis of the PLASE study, a prospective multicentre cohort study. Infants <30 weeks gestational age. 710 infants allocated to derivation and validation groups. **Score validated against:** Need for surgical ligation. **Statistical approach:** Logistic regression models were constructed to predict the future need for PDA surgery using three clinical and three echocardiographic indices measured at 3 days of age as candidate variables.	**Key findings** The model demonstrated high reliability, with ROC-AUC and ICC values of >0.8 and >0.9 for three echocardiographic measures and gestational age. **Conclusions** A surgical prediction model using simple indices at 3 days of age, and the validation data demonstrated good predictive ability. As 92% of the neonates undergoing surgical PDA ligation were treated with NSAIDs compared with 49% who did not receive surgery, this model appears to be at least somewhat sensitive to predict those neonates who will ultimately fail attempted pharmacologic treatment.	**PDA scoring system** Gestational age PDAd. LPAedv. LA/Ao. **Scoring**: Numeric model; higher scores predicted surgical PDA.
Mitra *et al*, SMART PDA^29^	**Study design and population** Multicentre, open-labelled, parallel-designed pilot randomised controlled trial. Preterm infants born <26 weeks of gestation with a PDA diagnosed within 72 hours after birth. 104 infants randomised to no treatment of the PDA in the first week of age vs **selective early pharmacological treatment** (SMART-PDA strategy) of a moderate-severe PDA shunt. SMART-PDA identified based on predefined clinical signs and routine screening echocardiography within the first 72 postnatal. Used ibuprofen as pharmacotherapy option over paracetamol. **Score validated against: No predefined outcome.** **Statistical approach:** **Aims:** Feasibility outcomes and clinical outcomes such as mortality, severe intraventricular haemorrhage, procedural PDA closure, chronic lung disease, NEC, ROP, sepsis, pulmonary haemorrhage.	**Key findings** Median age at treatment in the SMART arm was 2 days of age. 24% of infants in the SMART arm were not treated. With regards to patient-important outcomes (NEC, sepsis, pulmonary haemorrhage, ROP), the SMART strategy had an 83% posterior probability of a better WIN ratio in the first 7 days. **Conclusions** SMART strategy is feasible and led to improved outcomes (pulmonary haemorrhage/sepsis/ROP/NEC) compared with conservative arm in the first week of age.	**PDA scoring system** Combination of clinical signs (one or more) and echocardiographic markers: Defined as mild, moderate or severe dependent. **Clinical parameters** Need for supplemental oxygen. Non-invasive respiratory support or mechanical ventilation with MAP. Oliguria (urine output <1 mL/kg/hour) not explained by other clinical conditions (medications; birth asphyxia). Systemic hypotension (mean BP<gestational age in weeks at birth). Profound or recurrent pulmonary haemorrhage. Acute renal failure (oliguria with elevated creatinine levels). Haemodynamic instability requiring >1 cardiotropic agent. **Echocardiographic markers:** PDA size and shunt direction. Pulmonary and systemic shunt effects. LA/Ao. Transductal peak systolic velocity. Left ventricular output (mL/kg/min). Diastolic flow pattern in the descending aorta. **Scoring:** composite score with severity threshold for intervention within RCT.

BP, blood pressure; CLD, chronic lung disease; DFR, descending aorta flow reversal; E/A, ratio of early (E) to late/atrial (A) ventricular filling velocities; hsPDA, haemodynamically significant PDA; ICC, Intraclass Correlation Coefficient ; IVRT, Isovolumic Relaxation Time; LA/Ao, left atrium-to-aortic root ratio; LPA, left pulmonary artery; LPAedv, Left Pulmonary Artery end-diastolic velocity; LV, left ventricular; LVEDD, left ventricular end-diastolic diameter; LVO, left ventricular output; MAP, mean airway pressure; NEC, necrotising enterocolitis; NSAID, non-steroidal anti-inflammatory drug; PDA, patent ductus arteriosus; PDAd, ductus arteriosus diameter ; PDAsc, PDA Severity Score; PLASE, Proactive Diagnosis and Tailor-Made Treatment of Patent Ductus Arteriosus in Very Preterm Infants; PVL, periventricular leukomalacia; PV V_d_, pulmonary vein diastolic velocity (or D-wave); ROC-AUC, Receiver Operating Characteristic - Area Under Curve; ROP, retinopathy of prematurity; RPA, right pulmonary artery; SMA, superior mesenteric artery; SMART-PDA, Selective early medical treatment of the patent ductus arteriosus; SZMC, Shaare Zedek Medical Centre; TnECHO, targeted neonatal echocardiography .

### Trial setting and centres

- Include centres with readily available haemodynamic and echocardiographic expertise. While this may reduce generalisability to other centres, if benefit is shown in this setting, this provides the evidence-base to strengthen haemodynamic skills capacity across more centres.
- Centralised ‘ECHO Core’ to standardise the adjudication of haemodynamic significance, such as in the PIVOTAL trial (NCT05547165). Most RCTs and observational studies have relied on routine echocardiography services, a likely source of imaging and measurement variability.

### Intervention

- Unclear comparative risks and benefits of pharmacological agents (ibuprofen, indomethacin, acetaminophen); need head-to-head trials or strong meta-analyses comparing agents, doses and sequences across preterm gestational groups.
- Given the uncertain efficacy and risks of pharmacotherapy, alternative intervention including transcatheter closure, beyond COX and POX inhibitors and combination therapy should be explored.

### Considerations for timing

- The optimal and/or safe duration of “watchful expectant management” needs exploration. Although expectant management has not been proven harmful,^6 7^ follow-up of patients with non-documented ductal closure is advised.
- Efficacy of pharmacotherapy is influenced by the intervention timing.^2^
- Echocardiography screening and intervention should occur prior to the outcome, for example, within the first 24 hours to reduce IVH.
- We suggest 7–14 days for ‘later’ intervention; supported by the secondary analysis of both the PDA-TOLERATE^10^ and TRIOCAPI (Early Targeted Ibuprofen Treatment of Patent Ductus Arteriosus in Premature Infants)^31^ trials which demonstrated that prolonged exposure to a moderate-large PDA shunt was associated with an increased risk of BPD in infants who received mechanical ventilation ≥10 days.

### Comparator

- Specific practices in the ‘expectant’ arm have not been standardised and reported which may lead to variance across trials.^3^ This is problematic as some elements of the ‘expectant’ arm (eg, fluid restriction, diuretics, steroids, permissive hypercapnia, higher haematocrit and targeted oxygen saturations) may pose independent risk. The high proportion of open-label pharmacotherapy use in the control/expectant arm in some RCTs may have also reduced the distinct difference in exposures between the two groups, diluting any differences in the effect of the intervention.^6^
- A stringent protocol for expectant management and threshold for open-label use of the intervention are needed. Details of ventilator support, diuretics, postnatal steroids should be reported.

## Outcomes

The outcomes chosen should be important to both clinicians and families.

### Considerations for the choice of primary and secondary outcome measures

#### Early intervention (<24 hours)

Observational data shows that a very early (<24 hours) selective approach to infants with a moderate-high volume PDA shunt, using intravenous acetaminophen, decreases the rate of grade III/IV IVH, pulmonary haemorrhage and NEC without major adverse effects.^4^ In addition, patients randomised to early targeted PDA treatment in the recently concluded selective early medical treatment (SMART) PDA pilot trial of 116 infants born less than 26 weeks GA, showed that infants randomised to an early targeted approach had 85% probability^32^ of a better Win ratio with regards to patient-important outcomes (mortality, pulmonary haemorrhage, sepsis, NEC and severe ROP) compared with controls. Once again, it is important to highlight that these data are hypothesis generating; however, they provide biological justification for testing this approach in a larger RCT design. On the contrary, a recent Bayesian metanalysis of five trials where the open-label treatment rate was <25% showed a concerning signal towards increased risk of death and/or BPD further emphasising the potential dangers of pharmacotherapy.^33^ Of note, very few infants born <24 weeks GA, the population at greatest risk, have been enrolled in PDA trials; therefore, findings of published RCTs are not generalisable to the most immature infants.^6^ In summary, for very early intervention (<24 hours) PDA RCTs, grade III/IV IVH, pulmonary haemorrhage and mortality should be key outcomes.

#### Later (7–14 day) intervention

Ideally, the outcomes for later (7–14 day) intervention should be related to factors that drive clinicians to intervene; (1) minimising effects of sustained pulmonary overcirculation (eg, need for respiratory support); (2) intolerance and/or adverse effects from expectant management strategies (eg, heart dysfunction/failure); and (3) minimising effects of systemic hypoperfusion (eg, feeding intolerance, intestinal perforation/NEC, renal impairment).

### Selection of primary and secondary outcomes

Although there is evidence relating BPD to prolonged PDA exposure,^5^ clinicians’ decision to treat the PDA is not based solely on reducing BPD, which diminishes its relevance. In addition, BPD is an outcome defined by receipt of treatment at an arbitrary point in time (36 weeks postmenstrual age), rather than an objective diagnostic test. The relevance for a 22-week infant, who must wait 14 weeks to reach this threshold versus a 28-week infant who only waits 8 weeks is questionable.

For a more precise analysis of differences in outcomes, we propose the primary outcome as duration of invasive and/or non-invasive ventilation, with death as a competing risk. Secondary outcomes should include (1) key intermediary outcomes, such as moderate and severe BPD and chronic pulmonary hypertension,^5^ (2) parent-important measures, such as brain injury, NEC, sepsis, retinopathy of prematurity and (3) quality-of-life indicators such as long-term neurodevelopmental outcomes and respiratory morbidity.^34^ Table 3 provides a summary of the knowledge gaps and research priorities.

**Table 3:** Current knowledge gaps, research opportunities and priorities in patent ductus arteriosus management for preterm infants

PICO focus areas	Key barriers and challenges	Research opportunities and priorities
Population selection	No universally accepted definition of *haemodynamically significant* PDA with a moderate-to-high volume shunt.	Develop, validate and standardise composite echocardiography/clinical scoring systems (including metrics of pulmonary overcirculation, left ventricular loading conditions, systemic hypoperfusion) to stratify shunt severity. Infants with haemodynamic phenotypes, where PDA treatment may be harmful (eg, heart dysfunction, pulmonary hypertension) should be excluded.
	Variability/measurement error in echocardiography and interobserver differences.	Use centralised ‘ECHO core labs’ or standardised training/adjudication strategies in multicentre trials and test reproducibility and reliability of echocardiographic measures.
	Poor ability to predict which PDAs will persist and not close spontaneously (ie, population that will benefit from intervention).	Identify and validate predictors (clinical, imaging, biomarkers) of spontaneous closure vs persistence, ideally early (within first days).
	Limited data in extremely preterm infants <24 weeks.	Design trials that specifically include the most immature infants and specifically <24 weeks with appropriate adapted protocols and safety monitoring.
Intervention	Unclear comparative risk/benefit of different pharmacological agents (ibuprofen, indomethacin, acetaminophen, newer agents).	Head-to-head trials (or well-designed meta-analyses) comparing different agents, dosing regimens and sequences in different preterm gestation groups.
	Uncertainty of the role of currently available pharmaceutical agents, given their variable effectiveness and potential harmful effects. Even with PDA scores that can detect PDA with high volume shunt burden, pharmacotherapy is not 100% effective at closing the PDA.	Investigate alternative therapeutic agents (eg, transcatheter PDA closure) to cyclooxygenase and peroxidase inhibitors.
	Uncertainty about optimal timing of intervention (very early, early, late or deferred). No large multicentre trial of very early (<24 hours) intervention in the highest risk patients with the most pathological shunts.	Design trials comparing interventions at different time points (eg, <24 hours, 7–14 days, beyond) with appropriate stratification.
	Limited knowledge and ability to predict which infants are responders vs non-responders to pharmacotherapy.	Enhance prediction of responders to pharmacotherapy using predictive analytics including machine learning. This will help select which patients with a hsPDA will likely respond to, and benefit from pharmacotherapy vs waiting for definitive closure.
	Role and timing of catheter/device closure vs pharmacotherapy management vs expectant management.	Investigate the efficacy, safety and long-term outcomes of transcatheter closure (especially in low birthweight) and define criteria for when, and in whom, procedural closure is optimal.
Control	Lack of well-defined expectant (control) management protocols which are applied consistently across trials and the frequency of specific interventions that may have independent harm (eg, intravenous steroids) reported.	Define and standardise a protocol for ‘conservative management’ (fluid strategy, ventilator settings, diuretics, nutrition, steroids) in trials to reduce heterogeneity and limit open-label crossover.
Outcome	Incomplete evidence on long-term safety and developmental outcomes of PDA therapies.	Ensure extended follow-up (neurodevelopment, lung function, cardiac outcomes) in all trials of pharmacological and interventional closure.
	Uncertainty in the ideal outcome measures. Questionable relevance of BPD, given the diversity of the underlying phenotypes in BPD (eg, isolated lung disease, pulmonary hypertension, residual shunt physiology, hypertension associated left heart diastolic dysfunction and pulmonary vein disease). Important to incorporate short, medium and long-term outcomes that are meaningful to parents.	Work together with parents to co-develop a core outcome dataset for PDA trials.

BPD, bronchopulmonary dysplasia; hsPDA, haemodynamically significant PDA; PDA, patent ductus arteriosus.

### Trial methodological design

Intervention of an active management care pathway lends itself to both an infant-level or a step-wedge cluster RCT design.^35^ The latter involves random and sequential crossover of centres from control to intervention arm until all neonatal units in the trial have implemented the active pathway. However, a major drawback of a cluster RCT is the inability to fully control for variation in neonatal unit practices and the associated loss of statistical power due to intracluster correlation. As entire units rather than individual patients are randomised, outcomes within clusters are correlated and RCT design typically requires larger sample sizes and may still be influenced by between-unit practice variability. Sample size calculations and analysis must also address the confounding effect of time. Equipoise is an essential prerequisite, and open-label treatment in the expectant arm should be avoided.

## Conclusion

In summary, this review emphasises that although early PDA treatment trials show no clear benefit and even potential harm, these findings may not be generalisable to higher risk infants with prolonged exposure to high-volume shunts, nor interpreted to mean that the PDA is never clinically important. The question of whether a subgroup of infants, particularly the highest risk infants born under 28 weeks GA, could benefit from definitive ductal closure with low toxicity interventions remains uncertain and needs further evaluation.

## Supplementary material

10.1136/archdischild-2025-330266 online supplemental file 1
