# Supplementary material for: Making sense of the evidence: designing randomised controlled trials for preterm infants with high-shunt volume patent ductus arteriosus
Source: Arch Dis Child Fetal Neonatal Ed. 2026 Apr 27;111(5):e330266. doi: 10.1136/archdischild-2025-330266 (PMC13539860; doi:10.1136/archdischild-2025-330266)

Supplementary file

1) Supplementary Figure 1: ROBINS-I assessment of observational studies

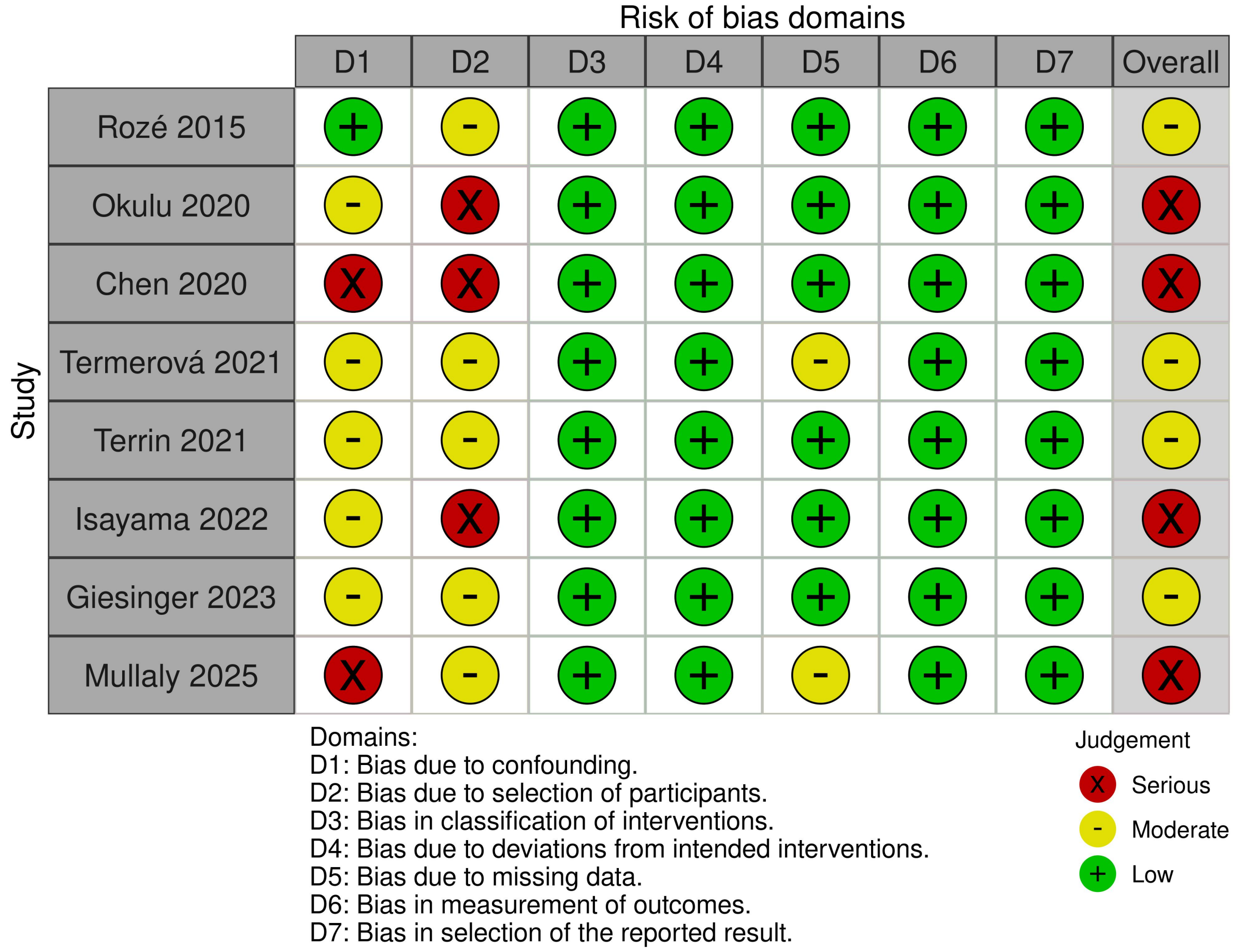

2) Supplementary figure 2 Risk of bias

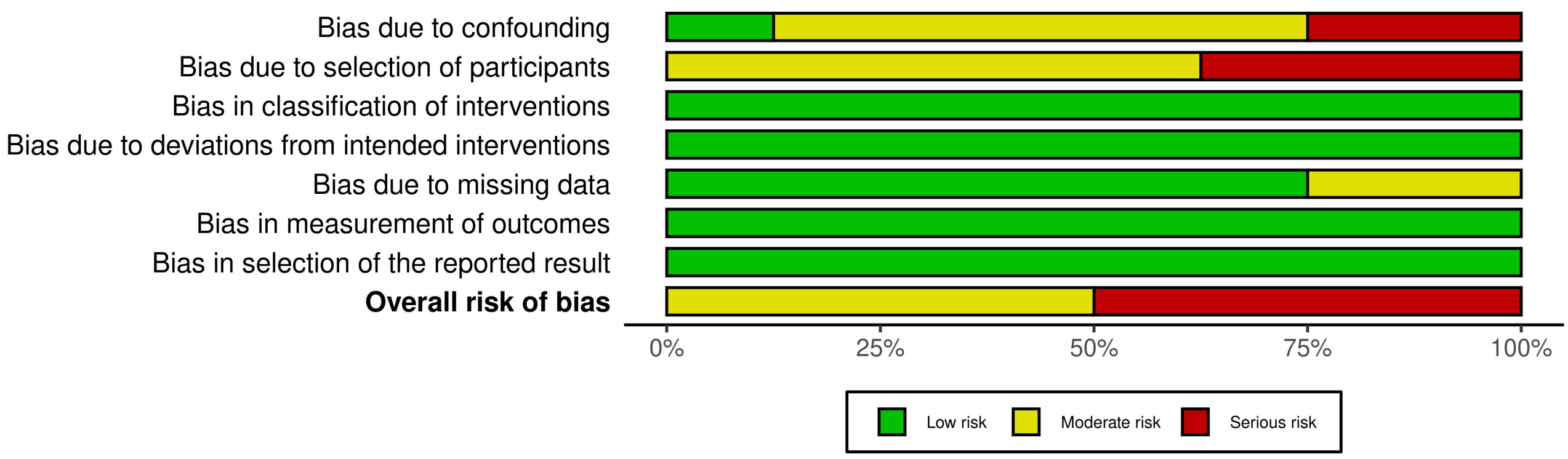

Supplement: Supplementary file 1 [file fetalneonatal-111-5-s001.pdf]
